# Supplementary material for: Automated Mass Photometry of Adeno-Associated Virus Vectors from Crude Cell Extracts
Source: Int J Mol Sci. 2024 Jan 9;25(2):838. doi: 10.3390/ijms25020838 (PMC10815086; doi:10.3390/ijms25020838)
Supplement: Supplementary file 1 [file ijms-25-00838-s001.zip › ijms-2777391-supplementary.pdf]

Supplementary Materials

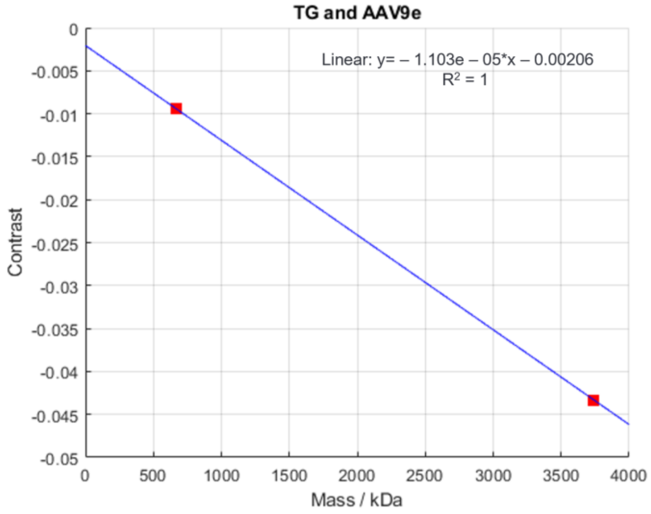

(a)

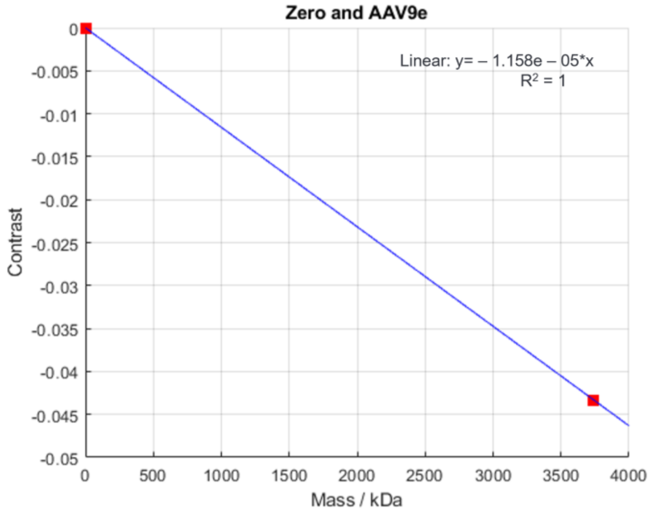

(b)

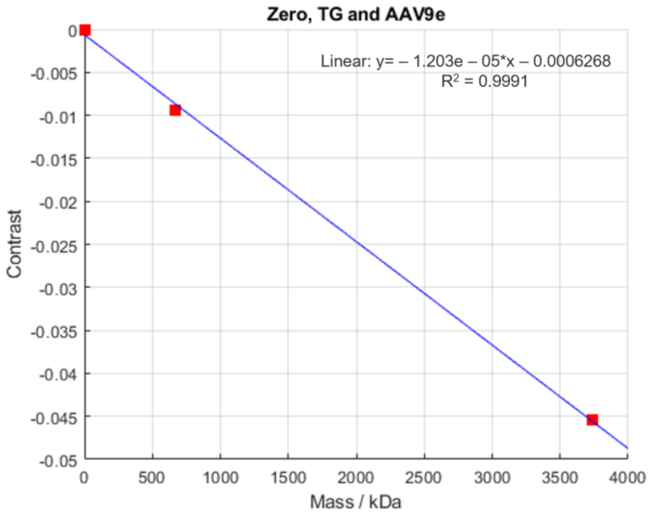

(c)

Supplementary Figure S1. Calibration curves obtained with (**a**) TG and AAV9e, (**b**) Zero and AAV9e and (**c**) Zero, TG and AAV9e.

Supplementary Table S1. Determination of the AAV subpopulations of an AAV9 sample consisting of ~33% empty, ~6% partially filled, ~52% full, and ~8% overfull particles under manual operation of the mass photometer using three different calibrations. x: two data points-based calibration with TG and AAV9e; y: two data points-based calibration with the zero point and AAV9; z: three data points-based calibration with the zero point, TG and AAV9e. E, PF, F and OF stand for empty, partially filled, full and overfull, respectively.

	AAV subpopulations							
**Calibration**	**Average E / %**	**RSD / %**	**Average PF / %**	**RSD / %**	**Average F / %**	**RSD / %**	**Average OF / %**	**RSD / %**
x[1)]	40.4	5.5	7.0	9.3	50.5	3.4	2.1	16.8
y[2)]	40.6	5.2	7.4	6.7	50.1	3.7	1.9	16.6
z[3)]	41.9	5.1	10.1	7.3	46.5	3.8	1.5	20.5

[1)] TG and AAV9e, [2)] Zero and AAV9e, [3)] Zero, TG and AAV9e

Supplementary Table S2. Determination of the molar masses of empty and filled subpopulations and of the transgene size of an AAV9 sample consisting of ~33% empty, ~6% partially filled, ~52% full, and ~8% overfull particles under manual operation of the mass photometer using three different calibrations. x: two data points-based calibration with TG and AAV9e; y: two data points-based calibration with the zero point and AAV9; z: three data points-based calibration with the zero point, TG and AAV9e.

	Empty AAVs		Full AAVs					
Calibration	Average mass / kDa	RSD / %	Average mass / kDa	RSD / %	Calculated transgene size / kDa	RSD / %	Expected transgene size / kDa	Error / %
x[1)]	3736	0.2	5211	0.4	1476	0.8	1400	5.4
y[2)]	3735	0.2	5139	0.3	1403	0.6	1400	0.2
z[3)]	3544	0.2	4896	0.3	1351	0.7	1400	3.5

[1)] TG and AAV9e, [2)] Zero and AAV9e, [3)] Zero, TG and AAV9e

Supplementary Table S3. Determination of the AAV subpopulations of an AAV9 sample consisting of ~33% empty, ~6% partially filled, ~52% full, and ~8% overfull particles under automated operation of the mass photometer using either the "buffer-free" or "buffer-dilution" focusing option. E, PF, F and OF stand for empty, partially filled, full and overfull, respectively.

	AAV subpopulations							
	Average E / %	RSD / %	Average PF / %	RSD / %	Average F / %	RSD / %	Average OF / %	RSD / %
Buffer-free	38.6	13.8	8.7	14.9	48.9	5.8	3.8	50.1
Buffer-dilution	36.6	4.4	11.5	14.4	49.5	5.2	2.5	8.0

Supplementary Table S4. Determination of the molar masses of empty and filled subpopulations and of the transgene size of an AAV9 sample consisting of ~33% empty, ~6% partially filled, ~52% full, and ~8% overfull particles under automated operation of the mass photometer using either the "buffer-free" or "buffer-dilution" focusing option.

	Empty AAVs		Full AAVs					
	Average mass / kDa	RSD / %	Average mass / kDa	RSD / %	Calculated transgene size / kDa	RSD / %	Expected transgene size / kDa	Error / %
Buffer-free	3789	1.8	5230	1.9	1441	2.2	1400	2.9
Buffer-dilution	3566	1.0	4922	0.9	1355	0.9	1400	3.2

Supplementary Table S5. Determination of the binding count rates for "buffer-free" and "buffer-dilution" sample measurements using an AAV9 sample consisting of ~33% empty, ~6% partially filled, ~52% full, and ~8% overfull particles under automated operation of the mass photometer.

	Binding count rate	
	Binding events	RSD / %
Buffer-free	2836	66.8
Buffer-dilution	5426	16.9

Supplementary Table S6. Determination of the AAV subpopulations of an AAV9 sample consisting of ~33% empty, ~6% partially filled, ~52% full, and ~8% overfull particles under automated operation of the mass photometer. The sample was measured five times on three consecutive days (days 1-3). E, PF, F and OF stand for empty, partially filled, full and overfull, respectively. E, PF, F and OF stand for empty, partially filled, full and overfull, respectively.

	MP							
	Average E / %	RSD / %	Average PF / %	RSD / %	Average F / %	RSD / %	Average OF / %	RSD / %
Day 1	38.5	8.1	8.0	5.4	49.7	6.0	1.4	17.6
Day 2	37.9	7.7	7.3	9.9	51.9	4.1	2.7	18.5
Day 3	37.6	12.8	8.9	13.4	49.6	5.5	4.5	16.7

Supplementary Table S7. Determination of the molar masses of empty and filled subpopulations and of the transgene size under automated operation of the mass photometer. Results were generated by five repetitive measurements of an AAV9 sample (~33% empty, ~6% partially filled, ~52% full, and ~8% overfull AAV particles) on three consecutive days (days 1-3).

	Empty AAVs		Full AAVs						
	Average mass / kDa	RSD / %	Average mass / kDa	RSD / %	Calculated transgene size / kDa	Average transgene size / kDa	RSD / %	Expected transgene size / kDa	Error / %
Day 1	3785	0.5	5222	0.5	1437	1440	0.6	1400	2.8
Day 2	3788	1.2	5237	1.6	1448				
Day 3	3794	1.8	5228	1.7	1434				
